# Endoscopic vacuum therapy for esophageal perforation: a multicenter retrospective cohort study

**DOI:** 10.1055/a-2042-6707

**Published:** 2023-04-20

**Authors:** Joanna Luttikhold, Lisanne M. D. Pattynama, Stefan Seewald, Stefan Groth, Bernhard K. Morell, Christian A. Gutschow, Satoshi Ida, Magnus Nilsson, Wietse J. Eshuis, Roos E. Pouw

**Affiliations:** 1 Division of Surgery, Department of Clinical Science Intervention and Technology (CLINTEC), Karolinska Institutet, and Department of Upper Abdominal Diseases, Stockholm, Sweden; 2 Department of Gastroenterology and Hepatology, Amsterdam University Medical Centers, Amsterdam, the Netherlands; 3 Department of Surgery, Amsterdam University Medical Centers, Amsterdam, the Netherlands; 4 Amsterdam Gastroenterology Endocrinology Metabolism, Amsterdam, The Netherlands; 5 Centre of Gastroenterology, Klinik Hirslanden, Zürich, Switzerland; 6 Department of Gastroenterology and Hepatology, Universitätsspital, Zürich, Switzerland; 7 Department of Gastroenterology and Hepatology, Stadtspital Zürich, Zürich, Switzerland; 8 Department of Visceral and Transplant Surgery, University Hospital Zurich, Zurich, Switzerland

## Abstract

**Background** Endoscopic vacuum therapy (EVT) is a novel treatment for esophageal perforations. This study aimed to describe initial experience with EVT of esophageal perforations due to iatrogenic cause, Boerhaave syndrome, or other perforations not related to prior upper gastrointestinal surgery.

**Methods** Data from patients treated with EVT for esophageal perforation at five hospitals in three European countries, between January 2018 and October 2021, were retrospectively collected. The primary end point was successful defect closure by EVT, with or without the use of other endoscopic treatment modalities. Secondary end points included mortality and adverse events.

**Results** 27 patients were included (median age 71 years). The success rate was 89 % (24/27, 95 %CI 77–100). EVT failed in three patients: two deceased during EVT (septic embolic stroke, pulmonary embolism) and one underwent esophagectomy due to a persisting defect. Two adverse events occurred: one iatrogenic defect expansion during sponge exchange and one hemorrhage during sponge removal. Median treatment duration was 12 days (interquartile range [IQR] 6–16) with 1 sponge exchange (IQR 1–3).

**Conclusion** EVT is a promising organ-preserving treatment for esophageal perforations, with a success rate of 89 %. More experience with the technique and indications will likely improve success rates.

## Graphical abstract

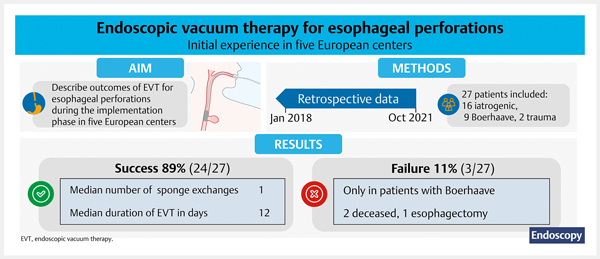

## Introduction

Esophageal perforations can be caused by iatrogenic or non-iatrogenic means, including endoscopic procedures and Boerhaave syndrome. Esophageal perforations often result in local inflammation and systemic sepsis, which is associated with severe morbidity and mortality rates varying from 12 % to 50 % 1 2 .

Endoscopic vacuum therapy (EVT) is a novel endoscopic approach for the treatment of transmural defects of the upper gastrointestinal (GI) tract, and is based on negative pressure wound therapy. For anastomotic leakage in the upper GI tract, EVT shows a success rate of between 60 % and 100 % 3 4 5 . Most studies are limited to small case series, which combine the data of esophageal perforations and anastomotic leaks.

The aim of this study was to describe the initial experiences with EVT for treatment of esophageal perforations not related to prior upper GI surgery.

## Methods

For this multicenter retrospective cohort study, we included all adult patients primarily treated with EVT for esophageal perforations, between January 2018 and October 2021, at five European tertiary referral centers. Presence of a perforation was based on computed tomography (CT) scan and/or contrast swallowing examination and/or an endoscopic finding of a defect in the esophageal wall.

Demographic and clinical data were retrospectively retrieved from electronic patient files.

The study was assessed by the local medical ethics committees, who waived the need for formal ethical review.

### Procedure

EVT procedures were performed under deep propofol sedation or general anesthesia. The sponge used was either an Eso-SPONGE (B. Braun Melsungen AG, Melsungen, Germany), or a self-fabricated sponge using a suction tube and absorbent wound dressing (Suprasorb CNP Drainage Film; Lohmann & Rauscher International, Rengsdorf, Germany).

The defect and any extraluminal cavity were cleaned during initial endoscopy. The EVT technique used was determined by the endoscopist after consideration of the defect and cavity widths and the extent of debris. Generally, patients with defects large enough for endoscope passage with large extraluminal cavities received intracavitary therapy and patients with small defects received intraluminal therapy. In some cases, intracavitary and intraluminal therapies were combined.

Under endoscopic visualization, the sponge was adequately positioned, and vacuum was applied at a negative pressure of –50 mmHg to –125 mmHg depending on the local standard operating procedure.

The frequency of sponge exchanges varied from 1 day to 1 week, depending on the center. In general, intracavitary sponges were exchanged more often than intraluminal sponges.

Cervical defects were not considered a contraindication to EVT as long as there was sufficient room to place a sponge to cover the defect without extending through the upper esophageal sphincter.

### Outcome variables

The primary outcome variable was successful defect closure by EVT, with or without the use of other endoscopic treatment modalities. Defect closure was determined by presence of at least two of the following criteria:

- endoscopic inspection: no remaining visible defect after treatment;
- imaging: no remaining contrast leakage on CT scan;
- oral intake: tolerance of oral intake after EVT.

Secondary outcome variables included success rate without the need for additional (non-)endoscopic treatment modalities, treatment characteristics, adverse events, incidents, and all-cause mortality, including in-hospital and 30-day mortality. Treatment characteristics were defined as outcomes specifically related to the EVT treatment, including treatment technique, number of sponge exchanges, and treatment duration. Adverse events were defined as any events interfering with the scheduled treatment 6 . Incidents were defined as unwanted events not interfering with the planned procedure. The Perforation Severity Score (range 0–18) was used to grade the initial defect, using 10 clinical variables 7 .

### Statistical analysis

Statistical analyses were performed using SPSS statistics software version 28 (IBM Corp., Armonk, New York, USA). Categorical outcomes were expressed as numbers with percentages and 95 %CIs, derived by binomial “exact” calculation. Continuous outcomes were expressed as median with interquartile range (IQR) owing to the limited sample size.

## Results

A total of 27 patients were included in the study. The characteristics and causes of perforation are listed in Table 1 .

**Table 1:** Baseline characteristics.

	Total (n = 27)
Age, median (IQR), years	71 (54–78)
Sex, n (%)	
Male	16 (59)
Female	11 (41)
Hospital, n (%)	27
Amsterdam UMC	16 (59)
Karolinska University Hospital	4 (15)
Klinik Hirslanden Zürich	4 (15)
Universitätsspital Zürich	2 (7)
Stadtspital Zürich	1 (4)
ASA score, n (%)	
1	2 (7)
2	10 (37)
3	12 (44)
4	3 (11)
Etiology of perforation, n (%)	
Iatrogenic	16 (59)
Boerhaave syndrome	9 (33)
Glass ingestion	1 (4)
Shot wound	1 (4)
Perforation Severity Score, median (IQR)	4 (3–7)
Perforation site, n (%)	
Cervical	2 (7)
Thoracic	25 (93)
Perforation size 1 , n (%)	25
Small (< 10 mm)	3 (12)
Intermediate (10–19 mm)	8 (32)
Large (≥ 20 mm)	14 (56)

IQR, interquartile range; UMC, University Medical Centers; ASA, American Society of Anesthesiologists.

1 The defect size was not documented in two patients.

### Primary outcome

**Fig. 1:**
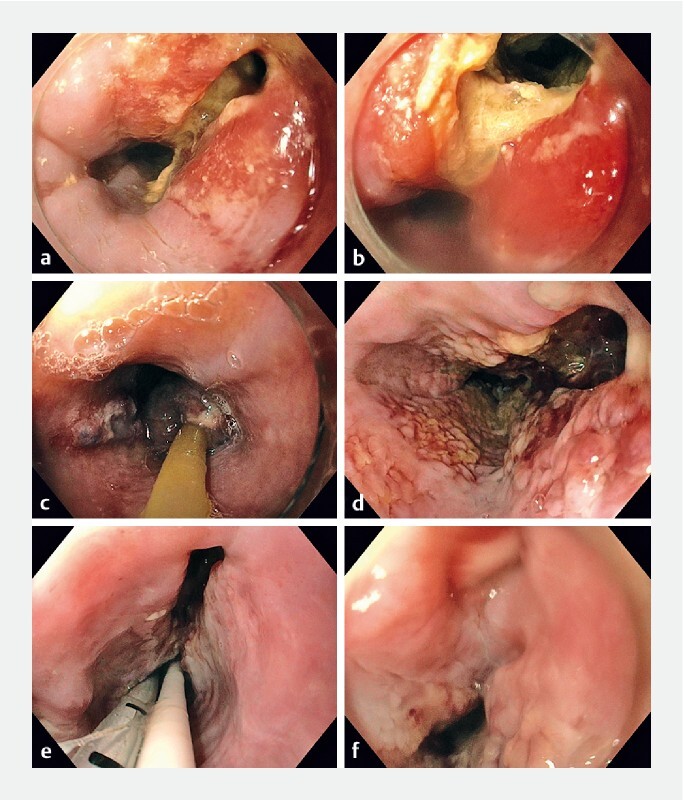
Trajectory of successful endoscopic vacuum therapy in a patient with Boerhaave syndrome. **a,b** The initial defect. **c–e** The defect during sponge exchange. **f** Successful closure of the defect.

Successful defect closure by EVT, with or without the use of other endoscopic treatment modalities, was 89 % (24/27, 95 %CI 77–100). Fig. 1 shows endoscopic images from successful EVT in a patient with Boerhaave syndrome.

EVT failed in three patients (11 %, 95 %CI 2–29). One patient died due to a septic embolic stroke after 4 days of EVT and another died due to a pulmonary embolism after 14 days of EVT. One patient underwent an esophagectomy because the defect showed no improvement. In this patient, the defect had been enlarged at the second EVT-related endoscopy and it was estimated that the sponge could not cover the defect adequately. Furthermore, in this case, EVT was initiated after considerable delay at the referring hospital, where the patient was treated for empyema, prompting the pursuit of a more vigorous approach.

### Secondary outcomes

Successful defect closure with EVT only was reached in 19/27 patients (70 %, 95 %CI 53–88). Additional treatment modalities were used in five patients. In one of the five patients, additional treatment was required owing to an inadequately collapsing cavity with EVT, and a muscle flap was subsequently placed into the cavity. After placement of the muscle flap, EVT was continued, resulting in successful closure of the defect. In four patients, a stent was placed after treatment and improvement of the defect with EVT, and left in situ for 1–4 weeks. In one patient, placement of a stent was decided because the sponge was dislodged repeatedly due to stenosis. In three other cases, the stent was placed over the small remaining defect at the discretion of the endoscopist in order to complete the last part of the treatment. Upon stent removal, all defects had closed successfully.

Endoscopic through-the-scope (TTS) clips were initially used in three patients and remained in situ for 1–5 days. However, due to insufficient treatment with TTS clips alone, EVT was initiated. Therefore, these cases were considered as being treated with EVT only.

All-cause mortality was 11 % (3/27, 95 %CI 2–29). Apart from the two aforementioned patients who died, a further patient died 8 days after completion of successful EVT, most likely due to a cardiac cause.

Two moderate adverse events occurred (7 %, 95 %CI 1–24): one defect enlargement caused by the scope during sponge exchange, and one hemorrhage during sponge removal, requiring blood transfusion. Two incidents occurred (7 %, 95 %CI 1–24): a small laceration at the site of the sponge during removal and epistaxis due to the sponge tube.

### Outcomes according to etiology

Outcomes according to etiology of the perforation are presented in Table 2 . All patients with unsuccessful EVT had Boerhaave syndrome.

**Table 2:** Outcomes according to etiology of perforation.

	Total (n = 27)	Iatrogenic cause (n = 16)	Boerhaave (n = 9)	Other (n = 2)
Success rate	27	16	9	2
Total, n (%)	24 (89)	16 (100)	6 (67)	2 (100)
EVT alone, n (%)	19 (70)	14 (88)	3 (33)	2 (100)
Perforation size, n (%)	25	15	9	1
Small ( < 10 mm)	3 (12)	2 (13)	0 (0)	1 (100)
Intermediate (10–19 mm)	8 (32)	7 (47)	1 (11)	0 (0)
Large ( ≥ 20 mm)	14 (56)	6 (40)	8 (89)	0 (0)
Perforation Severity Score, median (IQR)	4 (3–7)	4 (3–7)	7 (4–11)	4 (3–4)
Time to intervention, median (IQR), days	1 (0–3)	0 (0–3)	1 (1–4)	4 (2–6)
EVT technique, n (%)	27	16	9	2
Intraluminal	21 (78)	12 (75)	7 (78)	2 (100)
Intracavitary	3 (11)	2 (13)	1 (11)	0 (0)
Intraluminal and intracavitary	3 (11)	2 (13)	1 (11)	0 (0)
Additional treatment modalities, n (%)	5 (19)	2 (13)	3 (33)	0 (0)
Successful EVT, n	24	16	6	2
Sponge exchanges, median (IQR), n	1 (1–3)	1 (0–3)	2 (1–6)	1 (1–1)
Duration of EVT, median (IQR), days	12 (6–16)	13 (5–15)	15 (9–30)	13 (10–15)
Additional percutaneous and/or surgical drainage, n (%)	7 (26)	3 (19)	4 (44)	0 (0)
Hospital stay 1 , median (IQR), days	18 (11–34)	18 (11–31)	20 (9–52)	13 (11–14)
All-cause mortality, n (%)	27	16	9	2
In-hospital	3 (11)	1 (6)	2 (22)	0 (0)
30-day	0 (0)	0 (0)	0 (0)	0 (0)

EVT, endoscopic vacuum therapy; IQR, interquartile range.

1 Time to discharge or death.

## Discussion

This study describes the initial experiences with EVT for esophageal perforations, including Boerhaave syndrome and iatrogenic causes in five centers in the Netherlands, Switzerland, and Sweden. The rate of successful defect closure by EVT, with or without the use of other endoscopic treatment modalities, was 89 %, suggesting that EVT is an effective organ-sparing therapeutic option for these perforations, allowing invasive surgery to be avoided in the vast majority of patients. All-cause mortality and adverse event rates were low (11 % and 7 %, respectively).

These results correspond with previous studies on EVT in the upper GI tract, with success rates ranging from 60 % to 100 %, and an overall mortality rate of 0 % to 10 % 3 5 8 9 . However, the available literature on the topic mostly reports on results of EVT for anastomotic leakage and does not separately assess esophageal perforations. Furthermore, 30-day mortality rate of esophageal perforations in the literature appears higher, varying from 12 % to 50 % depending on the clinical status of the patient and the treatment technique used 1 2 .

As demonstrated in the current study, the success rate increased when EVT was combined with other treatment modalities. In this cohort, five patients were additionally treated with an intraluminal stent or surgical intracavitary placement of a muscle flap. Three patients received a stent over a small remaining defect, at the endoscopist’s discretion. It is likely that these defects would have also closed with EVT alone, as good clinical and endoscopic recovery was seen with EVT.

Although initial stent placement often causes a necrotic cavity to be sealed off, warranting additional percutaneous drainage, it may be a valid option if the defect is clean and shows good healing tendency after initial EVT, as it allows for the rest of the defect to heal while sealing the defect. Stent treatment allows the possibility of oral intake and requires fewer endoscopies compared with EVT with sponge exchanges. Future studies should focus on the optimal timing for combining EVT and stenting. In this respect, the recently released VACStent (MICRO-TECH Europe GmbH, Düsseldorf, Germany) may also be interesting. This device combines the sealing effect of a stent with the benefits of negative pressure wound therapy at the site of the defect, while keeping the stent in place with the vacuum and allowing for oral intake 10 11 .

In addition, one patient had a stiff pleura, causing inadequate expansion of the lung and collapse of the cavity with EVT alone. Therefore, this patient underwent surgical decortication with placement of a muscle flap into the pleural cavity. Given that EVT only works if the cavity collapses adequately, this could have been an exclusion criteria for EVT. However, this operation, in combination with endoscopic management, still has benefits when compared with an esophagectomy, as continuity of the gastrointestinal tract can be preserved. The optimal timing for such a surgical intervention has yet to be determined. One of the advantages of performing this surgery after initial EVT, is that the area in which the operation takes place is relatively clean and the patient is in a better clinical condition than at the start of the treatment, optimizing healing and reducing the risk of infection. Furthermore, thoracoscopic decontamination of the mediastinum could improve the healing process if significant decontamination is present.

Until recently, surgical treatment provided the best chance of cure for esophageal perforations. For example, Harikrishnan et al. (2020) described a retrospective overview of treatments for Boerhaave syndrome in a tertiary care center between 2008 and 2019 12 . In their study, 15/16 patients with Boerhaave syndrome underwent an esophagectomy, resulting in loss of continuity. However, with EVT, an esophagectomy could be prevented and gastrointestinal continuity maintained, which can be considered a big gain for patients’ quality of life. In the current cohort, only one patient underwent an esophagectomy.

Comparison of successful and unsuccessful groups may allow factors contributing to treatment failure to be identified and the best indications for EVT to be determined. However, given the relatively small and heterogeneous cohort in the current study, multivariable analysis on factors associated with successful or unsuccessful EVT could not be performed. Data did show that patients with a higher Perforation Severity Score were more likely to experience EVT failure. Moreover, success rates in patients with Boerhaave syndrome were lower (67 %) compared with patients with an iatrogenic perforation (100 %). This may probably be explained by the larger defect size and longer time to intervention in patients with Boerhaave syndrome, resulting in more leakage into the mediastinum and more systemically ill patients. Despite the lower success rates, EVT seemed to be an efficient and feasible therapy, even for Boerhaave syndrome.

Furthermore, perforations occurring during endoscopic treatment can often be adequately treated by primary closure with clips 13 . In such cases, EVT might be of additional value if a defect is too large for primary clip closure, if clip placement is technically not feasible due to a difficult location, or if there is persistent leakage despite placement of clips.

This study has several strengths. First, to our knowledge, this is the largest cohort describing the outcomes of EVT for exclusively esophageal perforations, excluding anastomotic leaks. Second, the international multicenter design allowed accumulation of expertise due to participation of multiple experts on EVT.

This study also has a number of limitations that need to be acknowledged. First, as this was a retrospective observational study and the EVT protocol differed per center, selection and information bias could have occurred during case and data retrieval. In addition, no comparison was made with a control group treated with other treatment modalities, such as placement of an intraluminal stent. Furthermore, due to the relatively small sample size, assessment of factors associated with EVT failure (e. g. the intracavitary versus intraluminal technique), could not be performed.

This paper retrospectively describes an international multicenter patient cohort treated with EVT for esophageal perforations, including those associated with Boerhaave syndrome and iatrogenic causes. Efficacy was established, with a success rate of 89 %. Although EVT for esophageal perforations is a promising technique promoting defect closure, a number of relevant questions remain, mostly regarding appropriate indications and combination with other treatment modalities.
